# SARS-CoV-2 infection and overactivation of Nlrp3 inflammasome as a trigger of cytokine “storm” and risk factor for damage of hematopoietic stem cells

**DOI:** 10.1038/s41375-020-0887-9

**Published:** 2020-06-01

**Authors:** Mariusz Z. Ratajczak, Magda Kucia

**Affiliations:** 1 Stem Cell Institute at James Graham Brown Cancer Center, University of Louisville, Kentucky, USA; 2 Department of Regenerative Medicine, Center for Preclinical Research and Technology, Medical University of Warsaw, Warsaw, Poland

**Keywords:** Cell biology, Stem cells

## Abstract

The scientific community faces an unexpected and urgent challenge related to the SARS-CoV-2 pandemic and is investigating the role of receptors involved in entry of this virus into cells as well as pathomechanisms leading to a cytokine “storm,” which in many cases ends in severe acute respiratory syndrome, fulminant myocarditis and kidney injury. An important question is if it may also damage hematopoietic stem progenitor cells?

Despite the massive virulence of SARS-CoV-2, we are in a much better position to investigate this virus than HIV 40 years ago, when the first cases of AIDS were reported. At the beginning of the HIV era it was still a conundrum how the virus could infect cells. It took some time to identify HIV co-receptors, and much more time was needed to develop antiretroviral drugs, and some of these could perhaps find therapeutic application today in SARS-CoV-2 patients before a safe and effective vaccine is available. To the benefit of mankind, more antiviral tools are available in the 21st century, and thanks to the internet it is much easier to exchange information in the fight with this invisible enemy.

Recently, *Leukemia* published a review on the role of the Nlrp3 inflammasome in normal and malignant hematopoiesis [1]. However, life (and nature) has unexpected twists and turns, and evidence has accumulated that the Nlrp3 inflammasome may be the culprit in certain complications of SARS-CoV-2 infection, which may affect several tissues and organs as well as potentially hematopoiesis. Specifically, there is evidence that the SARS-CoV-2 virus entry receptor (angiotensin-converting enzyme 2; ACE2) and receptor for angiotensin II (AT1) are expressed and functional on the surface of hematopoietic stem/progenitor cells (HSPCs) [2, 3]. Therefore, SARS-CoV-2 may on one hand directly infect pool of HSPCs, and on other pathological activation of Nlrp3 inflammasome may lead to cytokine storm and pyroptosis of these cells.

We already know that SARS-CoV-2 utilizes a spike protein (S) for attachment and entry into cells, which binds to a cell-surface expressed ACE2. Moreover, as reported recently, S protein must be primed by transmembrane protease serine 2 (TMPRSS2) to facilitate interaction with ACE2 and the subsequent fusion of viral and cellular membranes [4]. Therefore, some potential targets for future molecular interventions are already known.

Interestingly, while HIV sneaks into cells by employing entry receptors that are abundantly expressed on the surface of immune and hematopoietic cells (CD4, CXCR4, and CCR5), SARS-CoV-2 dysregulates the function of receptors involved in the regulation of blood pressure, fluid and electrolyte balance, as well as systemic vascular resistance [5]. Specifically, because SARS-CoV-2 utilizes the ACE2 receptor for cell entry, which becomes internalized after virus binding, it triggers hyperactivation of the renin–angiotensin–aldosterone system. To explain this complication, ACE2 is an enzyme that converts angiotensin I to angiotensin 1-9 and angiotensin II to angiotensin 1–7, and a lack of ACE2 leads to elevated levels of both of these peptides, which activate the angiotensin AT1 and AT2 receptors on the surfaces of endothelial, lung epithelium, intestine epithelium, kidney cells and what is important for us hematologists also on hemato/lymphopoietic cells [2, 3, 6]. Moreover, a lack of ACE2 impairs processing of angiotensin II to seven aminoacid peptides, angiotensin 1–7, which, by interacting with the MAS receptor, counteract the unwanted vasopressive and pro-fibrotic effects of the AT1 receptor [5].

Importantly, while considering the pathogenesis leading to initiation of a cytokine “storm” in the development of SARS-CoV-2 pathologies, one has to keep in mind the presence of a powerful proinflammatory system, the Nlrp3 inflammasome, which is expressed in many cell types, including innate immunity, endothelial, hematopoietic, lung epithelial, kidney, and cardiac cells [1, 7]. In fact, evidence indicates that the Nlrp3 inflammasome becomes activated in these cells in response to angiotensin II stimulation [8–11]. Whether interaction of the SARS-CoV-2 spike protein with ACE2 can do the same is currently being investigated in our laboratory. What is also important, our group demonstrated expression of the Nlrp3 inflammasome in hematopoietic stem/progenitor cells (HSPCs) [1] and what is also known ACE2 and AT1 receptors are expressed on HSPCs [2, 3]. Thus, determining the effect of SARS-CoV-2 on hematopoiesis requires careful investigation as these cells could be directly infected by virus and in addition a high level of angiotensin II could hyperactivate Nlrp3 inflammasome in these cells leading to cell death by pyroptosis. To support this angiotensin II mediated pyroptosis due to hyperactivation of Nlrp3 inflammasome has been already reported to occur in lung epithelium, kidney cells and cardiomyocytes [9–11].

It is known that activation of the Nlrp3 inflammasome triggers an immune response via intracellular caspase 1, which leads to (i) release of potent proinflammatory cytokines, such as interleukin-1β and interleukin 18, and (ii) by creating gasdermin D (GSDMD) pore channels in cell membranes, mediating the release of several biologically active danger-associated molecular pattern molecules (DAMPs). This initiates a sequence of events leading to amplification of the innate immune system response and activation of its major humoral arm, the complement cascade (ComC) [1]. In addition to DAMPs, the ComC, as recently reported, is directly activated by mannan-binding lectin (MBL), which binds to SARS-CoV-2 proteins [12]. Importantly, activation of the ComC via the MBL–MASP-2 protease complex leads, in parallel, to activation of the coagulation cascade (CoaC), and in patients infected with SARS-CoV-2, activation of coagulation correlates with a worse prognosis [13]. This explains why inhibition of the ComC or CoaC is considered to be a potential treatment option.

Considering the potential leading role of the Nlrp3 inflammasome hyperactivation in the pathogenesis of SARS-CoV-2 caused multi organ failure, we have to consider three scenarios for how this intracellular protein complex could become activated and finally leads to cytokine storm and cell death by pyroptosis (Fig. 1). First, it is possible that the SARS-CoV-2 spike protein (S), after binding to cell surface-expressed ACE2, directly triggers its enzymatic activation and downstream signaling. ACE2 has, in fact, been reported to be a signaling receptor. Thus, infection of cells could result in activation of Nlrp3 inflamamsome and pyroptosis. Second, also as previously reported, after binding to the AT1 receptor, angiotensin II is an important activator of the Nlrp3 inflammasome in lung, kidney cells, and cardiomyocytes. This could be a common mechanism in other cell types including hematopoietic cells. Third, recognition and interaction of the ComC with SARS-CoV-2 releases several potent cleavage fragments, such as the C3a and C5a anaphylatoxins as well as the C5bC9 membrane attack complex, which may also directly trigger activation of the Nlrp3 inflammasome. Several types of Nlrp3 inflammasome-positive cells, including innate immunity and lympho/hematopoietic cells express C3a- and C5a-binding receptors.

**Fig. 1:**
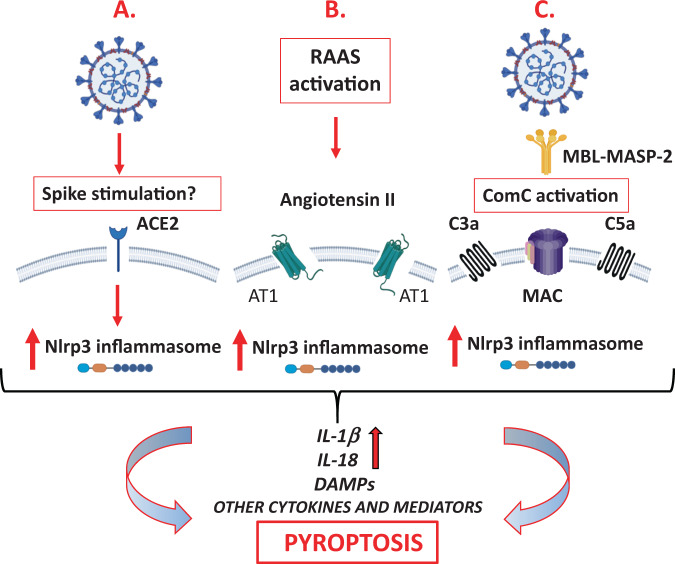
The pathways of Nlrp3 inflammasome activation in response to SARS-CoV-2 infection that may lead to initiation of cytokine storm and pyroptosis in cells including HSPCs. **a** It is possible that, by binding to ACE2 via the spike protein, SARS-CoV-2 directly activates the Nlrp3 inflammasome. This possibility is currently being investigated by our team. **b** Activation of the renin–angiotensin–aldosterone system (RAAS) leads to elevated levels of angiotensin II, which, after binding to the AT1 receptor, activates the Nlrp3 inflammasome in target cells. **c** Finally, the N proteins of the SARS-CoV-2 virus may activate the ComC in an MBL–MASP-2-dependent manner, and the ComC cleavage fragments (the C3a and C5a anaphylatoxins), as well as the C5b/C9 membrane attack complex (MAC), may additionally activate the Nlrp3 inflammasome in cells. (Of note, MASP-2 also activates the coagulation cascade by converting prothrombin into thrombin). Overall, activation of all three of these pathways leads to activation of caspase 1, the release of the mature IL-1β and IL-18 cytokines, the insertion of gasdermin D channels in the cell membrane, and the release of danger-associated molecular pattern molecules (DAMPs), which amplify the innate immune response and may lead to cell death by pyroptosis.

We postulate that the main problem with Nlrp3 inflammasome in pathogenesis of SARS-CoV-2 infection is its overactivation, leading to perturbation of mitochondrial function, the release of DAMPs, and cell death by pyroptosis [1, 7, 9–11]. This all raises the important question of whether Nlrp3 inflammasome inhibitors (e.g., the small molecule MCC950), similarly to other compounds proposed to affect SARS-CoV-2 binding to the cells and to inhibit intracellular virus amplification, and compounds that modulate activity of the innate immune system (e.g., ComC inhibitors) will find therapeutic application. There is also a potential role of mesenchymal stromal cells as potential modulators of the inflammatory response in the lungs and in other tissues.

An important question is whether SARS-CoV-2 infection affects stem cells including HSPCs. As mentioned above it is known that SARS-CoV-2 entry receptors are expressed on the surfaces of HSPCs, on endothelial progenitor cells, and even on more developmentally early common progenitors for the hematopoietic and endothelial lineages (manuscript in preparation). Moreover, since, as we already reported, HSPCs express Nlrp3 inflammasome components, it would be important to observe the long-term effects of SARS-CoV-2 infection on the hematopoietic stem cell compartment. In addition, virus could also affect stem cells in other organs, including lungs, myocardium, or small intestine. The complications of viral infection could be direct, involving specific entry receptors leading to elimination of infected cells, or indirect, involving activation of systemic immune response pathways. It is known that during first days of infection innate immunity is a main first responder against SARS-CoV-2 until acquired immunity responds with production of antibodies.

In conclusion, all pathological pathways depicted in Fig. 1 may lead to Nlrp3 inflammasome overactivation, implying that the virus not only triggers cytokine storm but most likely also affects the stem cell compartment in various organs and we may identify soon some remote consequences of SARS-CoV-2 infection [14, 15]. One of important questions remains if inhibition of overactivated Nlrp3 inflammasome could find clinical application?

However, to remain optimistic, let’s hope that the current armamentarium of scientific tools and the ability to rapidly communicate between different research groups will lead to dethroning of this treacherous virus, with its stolen crown (corona). We hope to soon banish this usurper from the realm along with its all of its relatives.
